# Multiscale Modeling and Simulation of Falling Collision Damage Sensitivity of Kiwifruit

**DOI:** 10.3390/foods13213523

**Published:** 2024-11-04

**Authors:** Yue Zhu, Licheng Zhu, Wenbei Wang, Bo Zhao, Zhenhao Han, Ruixue Wang, Yanwei Yuan, Kunlei Lu, Xuguang Feng, Xiaoxi Hu

**Affiliations:** 1 State Key Laboratory of Agricultural Equipment Technology, Beijing 100083, China; zhuyue920@gmail.com (Y.Z.); zhulicheng@caams.org.cn (L.Z.); wangwenbei66@163.com (W.W.); zhaoboshi@126.com (B.Z.); hanzhenhao@caams.org.cn (Z.H.); wangruixue@caams.org.cn (R.W.); 13241183263@163.com (K.L.); fxg725@163.com (X.F.); 2 Chinese Academy of Agricultural Mechanization Sciences Group Co., Ltd., Beijing 100083, China; 3 State Key Laboratory of Advanced Rail Autonomous Operation, Beijing Jiao Tong University, Beijing 100044, China; xiaoxhu@bjtu.edu.cn

**Keywords:** kiwifruit, falling collision, damage sensitivity, multiscale modeling, fem

## Abstract

Falling damage is the most common form of damage sustained by kiwifruit during the process of picking and post-processing, and it is difficult to conduct a quantitative analysis of this phenomenon through traditional experimental methods. In order to deeply understand the sensitivity of kiwifruit to falling collision damage, the finite element numerical simulation method was used to evaluate and predict the sensitivity of kiwifruit to falling collision damage during harvesting. First, we obtained the appearance characteristics of kiwifruit through reverse engineering technology and determined the geometric and mechanical property parameters of kiwifruit through physical mechanics experiments. Then, according to the characteristics of fruit tissue structure, a multiscale finite element model, including the skin, pulp, and core, was constructed to simulate the effects of different falling heights, collision angles, and contact surface materials on fruit damage, and the accuracy of the model was verified through falling experiments. Finally, based on the simulation results, the Box–Behnken design was employed within the response surface methodology to establish a sensitivity prediction model for the drop damage sensitivity of kiwifruit across different contact materials. The results showed that the maximum relative error between the speed change obtained using finite element simulation and the speed obtained by the high-speed camera was 5.19%. The model showed high rationality in energy distribution, with the maximum value of hourglass energy not exceeding 0.08% of the internal energy. On the contact surface material with a large elastic modulus, a higher falling height and larger collision angle will significantly increase the risk of fruit bruise. When the contact surface material was a steel plate, the falling height was 1 m, and the collision angle was 90°; the maximum bruise sensitivity of kiwifruit reached 6716.07 mm^3^ J^−1^. However, when the contact surface material was neoprene, the falling height was 0.25 m, and the collision angle was 0°, the damage sensitivity was the lowest, at 1570.59 mm^3^ J^−1^. The multiscale finite element model of kiwifruit falling collision constructed in this study can accurately predict the damage of kiwifruit during falling collision and provide an effective tool for the quantitative analysis of kiwifruit falling collision damage. At the same time, this study can also provide guidance for the design and optimization of the loss reduction method of the harvesting mechanism, which has important theoretical significance and practical value.

## 1. Introduction

Kiwifruit, as a highly nutritious fruit, is deeply loved by consumers. However, in the production process, such as picking and post-harvest treatment, kiwifruit is prone to bruises due to falling and collision. The bruising of fruit refers to the damage of the subcutaneous tissue of fruit caused by an external force, often without rupturing the skin, manifesting as the browning of the pulp tissue. The occurrence of bruising not only reduces the quality of fruit but also easily causes infection in other healthy fruits, which directly affects the economic benefits of fruit farmers [1,2]. Therefore, it is of great significance to study the mechanical behavior of kiwifruit in the process of falling collision to reduce fruit damage and improve fruit quality. Kiwifruit is classified as a post-ripening fruit, and the bruise characteristics produced by collision on the fruit are delayed and will not immediately appear. In addition, the pulp is relatively dark in color, making it difficult to directly observe and quantitatively analyze the degree of the bruise with the naked eye [3]. The traditional methods for detecting fruit bruises usually require removing the skin or slicing the pulp to directly observe the internal structure and color changes. However, in this method, the complete tissue structure of the fruit will be destroyed, resulting in a large error in the measurement results, which is not conducive to application in actual production [4].

With the development of non-destructive testing technology, non-destructive methods, such as nuclear magnetic resonance (NMR) and spectral imaging, have been widely used for fruit bruise detection and quantitative analysis. These technologies can track the wavelengths or infrared absorption that are sensitive to bruises and realize the rapid non-destructive detection of bruises by analyzing the spectral differences between damaged fruit and normal fruits in a specific wave band without changing the structure of the fruit [5,6,7]. Chigwaya et al. [8] used X-ray computed tomography (CT) and 3D image analysis technology to analyze the internal browning of Fuji apples after carbon dioxide stress. The results showed that the method can accurately identify and quantify the range and degree of browning inside apples. Razavi et al. [9] used magnetic resonance imaging (MRI) technology to quantitatively evaluate the damage volume of pears under static load. Through MRI scanning on pears under different load conditions, internal damage images of pear bruising were obtained. Ebrahimi et al. [10] used a convolution neural network and hyperspectral imaging technology to detect the bruise of kiwifruit, collected the reflectivity data of kiwifruit in different spectral ranges, and constructed a bruising recognition convolution neural network model to identify and distinguish damaged and undamaged kiwifruit. The results showed that this method can efficiently and accurately detect the bruising of kiwifruit, including a slight bruise that is imperceptible to the naked eye, while maintaining the integrity of the fruit and avoiding additional damage. Lü et al. [11] used hyperspectral imaging technology to detect the invisible bruises of kiwifruit and put forward an early hidden injury identification method based on hyperspectral imaging technology. The experimental results showed that the error of hyperspectral imaging in detecting recessive damage in fruit is 14.5%. However, the limited sample data may affect the generalization ability of the model. At the same time, high cost and operational complexity limited its popularization in small-scale agricultural production. Shao et al. [12] used near-infrared spectroscopy (Vis-NIR) reflection technology and a multivariate analysis method to judge the degree of cherry bruises and realized the effective identification and evaluation of cherry bruises. The experimental results showed that the accuracy of the classification of bruises reaches 93.3%. Although non-destructive detection methods, such as magnetic resonance imaging (MRI) and spectral imaging, can provide information on the internal structure, maturity, and quality of fruits and vegetables, it is difficult to popularize and apply them on a large scale due to the high cost of MRI equipment, slow scanning speed, limited resolution of spectral imaging, need to adjust parameters for different fruits, and poor effect of processing dark or high-light absorption fruits.

The finite element method (FEM) is an efficient numerical calculation method that solves complex engineering problems by approximately solving partial differential equations and has been widely used in predicting damage to agricultural products such as fruits and vegetables [13,14,15]. Hou et al. [16] determined the mechanical parameters of blueberries and built a multiscale calculation model using the finite element method to study the mechanical shock sensitivity of blueberries. The results showed that the established finite element model can effectively simulate and predict the damage of blueberries during falling, providing important guidance for the optimal design and post-harvest treatment of blueberry harvesting equipment. Xu et al. [17] evaluated the damage of white radishes under different impact conditions using the finite element method (FEM) and response surface methodology (RSM). By simulating the stress and strain distribution of white radishes under external force, the vulnerable areas were accurately predicted, and an empirical model for predicting the sensitivity of siltation was established. The results showed that the relative error between the predicted results of the model and the drop test results is less than 14.72%. Hou et al. [18] established a finite element model of mulberry fruit using the numerical simulation method and simulated the dynamic response of the fruit during falling. The stress distribution and energy absorption characteristics of the fruit at the moment of contact with different surface materials and the mechanism of mulberry fruit damage under different falling conditions were analyzed. The results showed that the numerical simulation results are in good agreement with the experimental observation results, and the finite element model can effectively stimulate the falling damage of mulberry fruit. An et al. [19] established a finite element model of strawberry falling damage based on the numerical simulation method of the dynamic finite element method and simulated the stress and strain field distribution of strawberries falling freely from different heights. It was found that there was a strong linear correlation between the internal damage and the surface damage of strawberry fruit. The predicted results of the model were in good agreement with the experimental data, which could accurately predict the damage of strawberries falling at different temperatures, and provided important theoretical support and practical application value for the control of post-harvest strawberry damage. In addition, Du et al. [20] obtained the geometric and mechanical parameters of kiwifruit through experiments, established a finite element model of kiwifruit’s falling damage, simulated the deformation and damage under different external forces, and verified the accuracy of the model by comparing the simulation results with the experimental data.

To sum up, although the non-destructive detection method has made some achievements in fruit bruise detection, its application in large-scale production is limited. Because of its high efficiency and accuracy, the finite element method has become an effective tool for studying fruit damage. However, at present, the systematic research on the multiscale damage behavior of kiwifruit during falling is still relatively scarce. The lack of research limits the in-depth understanding of the mechanism of kiwifruit fall damage and hinders the development of effective technologies and methods to reduce the mechanical damage of kiwifruit. Therefore, it is necessary to establish a multiscale finite element model that can accurately characterize the multi-component material properties of kiwifruit skin, pulp, and core to simulate and predict the damage of kiwifruit under different falling conditions. This will provide a solid theoretical basis for optimizing the post-harvest processing technology of kiwifruit and improving packaging and transportation conditions.

In this study, a multiscale finite element model of kiwifruit was established by combining experiments with numerical simulation, and its mechanical response during falling collisions was analyzed in depth to explore the sensitivity of fruit falling damage. Firstly, the geometric and mechanical parameters of kiwifruit components (skin, pulp, and core) were obtained through physical and mechanical experiments, which provided necessary data support for multiscale modeling. Based on this, a multiscale finite element model, including the skin, pulp, and core, was constructed to simulate the damage of fruit under different falling conditions. The accuracy of the model was verified using the drop experiment, and the accuracy and reliability of the model in predicting the fall damage of kiwifruit were evaluated. The effects of drop height, impact angle, and contact surface material on the sensitivity of kiwifruit fall bruises were systematically analyzed. Finally, based on the simulation results, a response surface method was used to establish a prediction model for the sensitivity of kiwifruit drop bruising sensitivity, which provided theoretical guidance and an optimization basis for the post-harvest treatment, packaging, and transportation of fruits. The main objectives of this study are as follows:

(1) Obtain geometric and mechanical parameters of kiwifruit components through experiments, providing the necessary material property data for multiscale finite element modeling.
(2) Construct a multiscale finite element model of kiwifruit, including the skin, pulp, and core, to improve the accuracy and reliability of simulation analysis of fall damage.
(3) Systematically analyze the key factors affecting the fall damage of kiwifruit, and reveal the mechanical mechanism of the damage evolution.
(4) Establish a sensitivity prediction model for kiwifruit drop damage, providing theoretical guidance and an optimization basis for the post-harvest treatment, packaging, and transportation of kiwifruit.

## 2. Materials and Methods

### 2.1. Materials and Equipment

**Figure 1:**
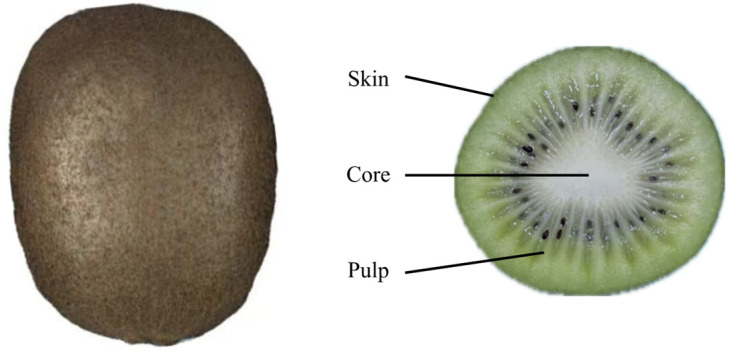
Kiwifruit samples.

In this study, the Xuxiang kiwifruit, which is the most widely cultivated and commercially valuable variety in China, was selected as the research object. The fruits were harvested in mid-October from an orchard in Meixian County, Shaanxi Province (latitude 34.16° N, longitude 107.75° E). At the time of harvest, the fruits had reached commercial maturity, with a soluble solids content of 7.0%. To ensure the reliability of the experimental results, all fruits were manually harvested using random sampling principles to avoid damage to the appearance and flesh tissue of the fruits during the harvesting process. To prevent moisture loss from the fruits, the harvested fruits were quickly transported back to the laboratory and stored in a constant temperature and humidity chamber (model LRH-500CA, Shanghai Yiheng Scientific Instrument Co., Ltd., Shanghai, China) at a temperature of 25 °C and relative humidity of 80%. Before the experiment, the samples were taken out and allowed to equilibrate at room temperature for 2 h before determining their physical and mechanical properties. All experiments were conducted under laboratory conditions and completed within 4 h. The Xuxiang kiwifruit samples used in the experiment are shown in Figure 1.

### 2.2. Measurement of Geometrical and Mechanical Properties of Kiwifruit

In this study, 60 kiwifruits with the same maturity, no mechanical damage, and similar sizes were selected as experimental samples. In order to ensure the reliability of the experimental results and the consistency of the data, the geometric size of each fruit was accurately measured before the experiment. With a vernier caliper with an accuracy of 0.01 mm, the length (*L*), maximum diameter (*D_max_*), and minimum diameter (*D_min_*) of the equatorial section of the fruit were measured, the measured geometric size data were statistically analyzed, and the average values of various geometric parameters were calculated. The mass (*m_a_*) and volume (*v_a_*) of kiwifruit were measured using the electronic balance (WT10002A, WANT Balance Instrument Co., Ltd., Changzhou, China; accuracy 0.01 g) and drainage displacement methods, respectively. According to the measured mass and volume, the density of the fruit was calculated using the formula ρ = *m_a_*/*v_a_*, which provided the necessary physical parameters for the subsequent mechanical analysis. Kiwifruit is mainly composed of skin, pulp, and a core, each with significantly different mechanical properties. To accurately characterize the mechanical property parameters of various parts of kiwifruit, tensile and compression experiments were conducted based on the ASABE compression test standard for food materials, ensuring the standardization of the experimental process and the validity of the data [21]. Key mechanical parameters, such as Young’s modulus, the tangential modulus, and the yield stress of the skin, pulp, and core, were determined using these experiments. Mechanical properties were tested using an electronic universal testing machine (DF22-102T, China Central Machinery Test Equipment Co., Ltd., Beijing, China). A circular compression plate with a diameter of 250 mm was used to conduct single compression tests on both the pulp and core of kiwifruit. The loading speed was set to 3 mm/min with a preset trigger force of 0.5 N. Loading was immediately stopped upon the occurrence of compression failure, and the retraction speed was set to 10 mm/min. The force-displacement curve during the compression process was recorded in real time using the machine’s built-in sensor to ensure accurate data acquisition. Due to the relatively thin skin of the kiwifruit, we designed a specialized tensile fixture to ensure the stable gripping of the skin samples during testing. The skin samples were gripped at both ends within the fixture and stretched at a rate of 3 mm/min until a fracture occurred. Samples with the fracture located in the mid-region were considered valid and were subjected to data recording and analysis. To enhance the reliability of the experimental data, each experimental group was repeated five times, with a total of five independent samples tested, and the average values were calculated as final results. According to Equations (1)–(3), the stress, strain, and modulus of elasticity values for the skin, flesh, and core were calculated.

(1) $$\rho =\frac{F}{S}$$

(2) $$\varepsilon =\frac{\Delta L}{L}$$

(3) $$E=\frac{\sigma }{\varepsilon }=\frac{\mathrm{FL}}{S\Delta L}$$

where *σ* is stress (MPa); *ε* is the tensile (compressive) strain (%); *E* is elastic modulus (MPa); *F* is the test load (N); *L* is the initial length (mm); *S* is the cross-sectional area of the material in the direction of tension (compression) (mm^2^); and ∆*L* is the elongation length (mm).

**Figure 2:**
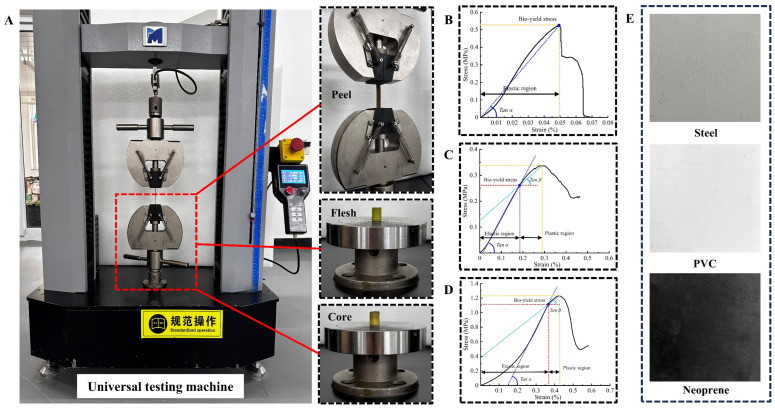
Measurement of mechanical parameters of fruits. (**A**) Electronic universal testing machine; (**B**) skin stretching; (**C**) pulp compression; (**D**) core compression; and (**E**) contact surface materials.

Data processing and analysis were conducted using Origin 2021 (Origin Lab, Northampton, MA, USA). The tensile stress–strain curves of the kiwifruit skin (Figure 2B), pulp (Figure 2C), and core (Figure 2D) were plotted. The results indicated that during the stretching process, the stress of the skin increased approximately linearly with the strain until it reached the biological yield point. Subsequently, the skin began to crack, leading to complete failure. In contrast, during the compression process, both the pulp and core exhibited elastic–plastic characteristics. When the stress exceeded the biological yield point, irreversible deformation occurred in the fruit structure, resulting in rupture or other microscopic damage.

### 2.3. Three-Dimensional Reverse Modeling of Kiwifruit

Kiwifruit has an irregular shape and complex surface, and its geometric characteristics are difficult to accurately describe using parametric modeling methods [22]. Traditional geometric modeling methods usually simplify fruits into regular geometric shapes for modeling and analysis. However, the simplification ignores the real geometric details of the fruit, resulting in a deviation between the finite element method results and the actual situation [23,24,25]. In order to solve the problem and improve the accuracy of the finite element model, reverse engineering technology was used in this study to model kiwifruit. Reverse engineering is a technology to reconstruct the digital model of existing objects by measuring and analyzing their shapes, sizes, and structures, which can accurately obtain the three-dimensional spatial information of objects and has been widely used in the three-dimensional modeling of complex biomaterials [26]. In the field of fruit mechanics, reverse engineering technology is helpful in establishing high-precision geometric models to improve the accuracy of simulation analysis [22,27]. According to the geometric dimension measurement and statistical analysis of the collected kiwifruit samples in the early stage, a kiwifruit whose length (*L*), maximum diameter of equatorial section (*D_max_*), the minimum diameter of equatorial section (*D_min_*) were all close to the average size of the sample was selected as the reverse modeling object, and the geometric dimension of the selected sample is shown in Table 1.

**Figure 3:**
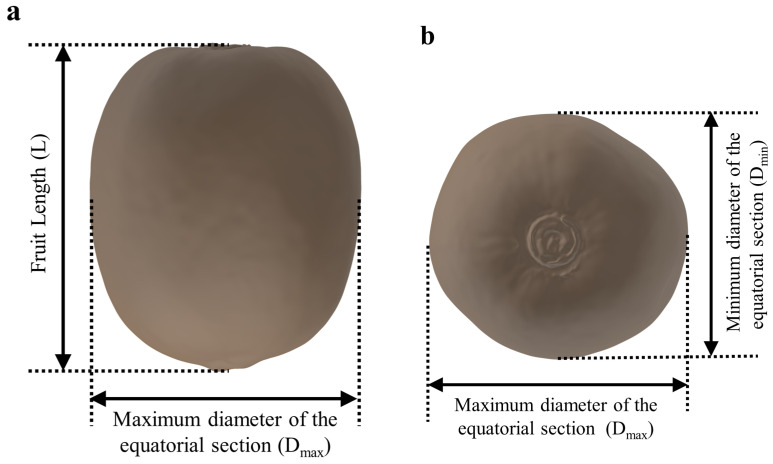
Reverse-engineered 3D geometric modeling of kiwifruit. (**a**) Front view of the model. (**b**) Top view of the model.

In order to accurately capture the complex surface details of kiwifruit, a high-precision 3D laser scanner (Solution IX Rex Can III, Seongbuk-Gu, Seoul, Republic of Korea) was used to collect point cloud data on the surface of kiwifruit. By denoising, smoothing, and surface stitching the point cloud data, a three-dimensional geometric model of kiwifruit with a smooth and continuous surface was constructed. Finally, using SolidWorks 2021 (Dassault Systems S.A., Waltham, MA, USA) three-dimensional modeling software, the internal structure of kiwifruit was further refined, and a multiscale three-dimensional geometric model of kiwifruit, including the skin, pulp, and core, was constructed. The reverse model of the fruit is shown in Figure 3.

### 2.4. Finite Element Modeling and Simulation

#### 2.4.1. Model Simplification

**Figure 4:**
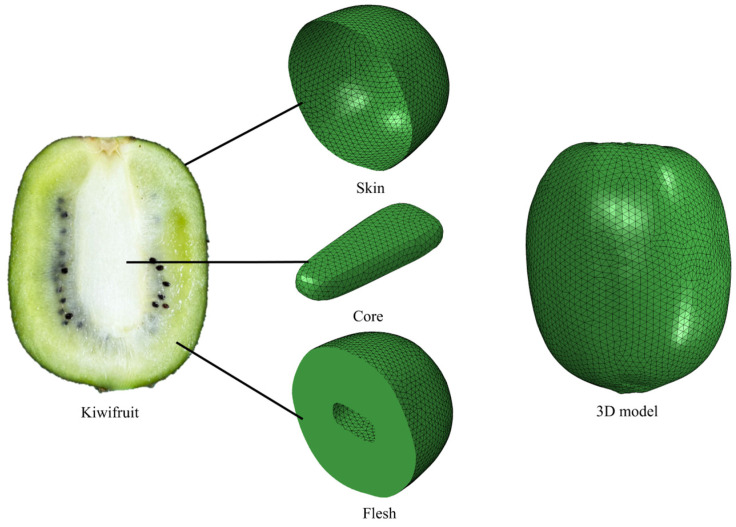
Fruit tissue structure and 3D model.

When studying the falling damage behavior of kiwifruit, the main focus is on the damage to pulp tissue. However, the structure of kiwifruit is complex. If all the internal details are modeled comprehensively, it will not only greatly increase the complexity of numerical simulation but also significantly increase the calculation cost and reduce the simulation efficiency. Therefore, to improve the feasibility of simulation and calculation efficiency, it is necessary to simplify the internal structure of fruit reasonably during model construction. According to the tissue structure characteristics of kiwifruit, the effects of secondary components, such as seeds, tissue fluids, and fibers, on the overall mechanical behavior of kiwifruit were ignored in this study, and the fruit was simplified into a multiscale geometric entity model, including the skin, pulp, and core. In addition, to further reduce the complexity of material constitutive equations and improve the convergence and stability of finite element calculation, the skin, pulp, and core were regarded as isotropic elastic–plastic materials in this study. Considering that the skin is relatively thin and plays a protective role in the overall structure of the fruit, the skin was constructed as a 0.3 mm-thick shell unit in the finite element model. The pulp and core were modeled in the form of solid units to accurately reflect their deformation and damage behavior when subjected to external forces. The simplified 3D geometric model is shown in Figure 4.

#### 2.4.2. Finite Element Simulation

**Figure 5:**
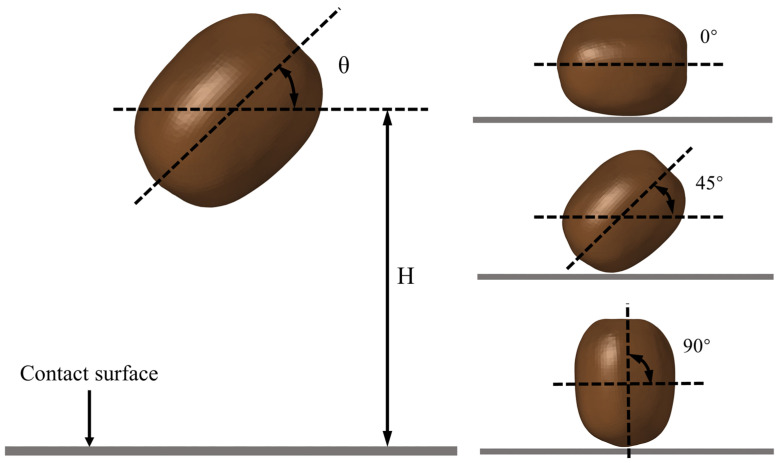
Schematic diagram of different drop heights and collision angles.

We investigated the effects of different falling heights, collision angles, and contact materials on the falling damage of kiwifruit. In this study, the finite element analysis software ABAQUS/Explicit v. 2021 (Dassault Systémes, Vélicy-Villacoublay, France) was used to simulate the finite element model of kiwifruit and analyze the results visually. By simulating the falling process of kiwifruit under different working conditions, the sensitivity of kiwifruit to falling damage was evaluated. In the simulation process, three main factors affecting the degree of kiwifruit fall damage were considered comprehensively: falling height, collision angle, and contact surface material [28]. In order to comprehensively evaluate the impact of these factors on the fruit falling damage, 27 different simulation scenarios were designed in this study, including three different falling heights (0.25 m, 0.5 m, and 1 m), three collision angles (0°, 45°, 90°), and three different collision interface materials (steel, PVC and neoprene), to cover the kiwifruit during picking, packaging, and transportation. The drop model is shown in Figure 5.

#### 2.4.3. Model Definition

In order to simulate the interaction of various components of kiwifruit during falling, different constraints were adopted in the model to ensure the linkage and consistency between components. The interaction between skin and pulp was bound through the “Tie” constraint to ensure that there would be no relative sliding or separation between them, thus more truly reflecting their deformation behavior during the collision. The “Embedded” constraint was adopted in the interaction between the core and the pulp, which can more truly describe the embedded relationship between the core and the surrounding pulp, reflecting the stress transmission and support of the core to the pulp under the action of external force. In order to improve the calculation efficiency and reduce the simulation time, the initial position of the kiwifruit was set close to the surface of the collision surface material, and the final speed of fruit falling was taken as the initial speed of the collision, directly simulating the dynamic process at the moment of the collision. The initial velocity can be calculated according to the formula of free fall ($v_{i}=\sqrt{2{\mathrm{gh}}_{i}}$), where *g* is the acceleration of gravity (9.8 m/s^2^) and *h_i_* is the initial falling height [29]. In the process of setting the solution time, factors such as the fruit deformation process, contact rebound period, and duration of collision energy absorption were comprehensively considered, and the simulation time of each scene was set to 0.02 s to ensure that the simulation could fully capture the dynamic response of kiwifruit in the process of falling collision and the reliability of the simulation results.

#### 2.4.4. Mesh Division

**Figure 6:**
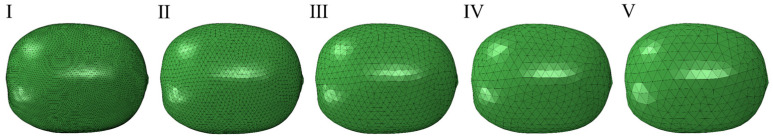
Kiwifruit mesh model.

In the finite element simulation calculation, it is a key factor to select the appropriate grid element size and type to ensure the calculation accuracy and efficiency. A reasonable grid division strategy can not only ensure the accuracy of simulation but also effectively save computing resources and improve the reliability of simulation results. In view of the irregular geometric shape of kiwifruit, the traditional hexahedral mesh generation method makes it difficult to generate high-quality meshes, and it is easy to produce distorted or twisted elements when fitting the complex contours of the fruit surface and interior, thus affecting the convergence of simulation and the accuracy of calculation results. In contrast, the tetrahedral element has good geometric adaptability, which can maintain the high quality and numerical stability of the model without significantly increasing the computational burden [30]. Therefore, in this study, a tetrahedral mesh generation strategy was adopted to mesh the three-dimensional finite element model of kiwifruit, with the mesh sizes and quantities shown in Table 2. In order to determine the optimal grid size, HyperWorks 2021 (Altair Engineering Inc., Troy, MI, USA) grid division software was used to generate five finite element models of kiwifruit with different grid sizes, and the grid sensitivity of the models was analyzed. The grid model is shown in Figure 6.

#### 2.4.5. Extraction of Bruising Volume from the Finite Element Model

**Figure 7:**
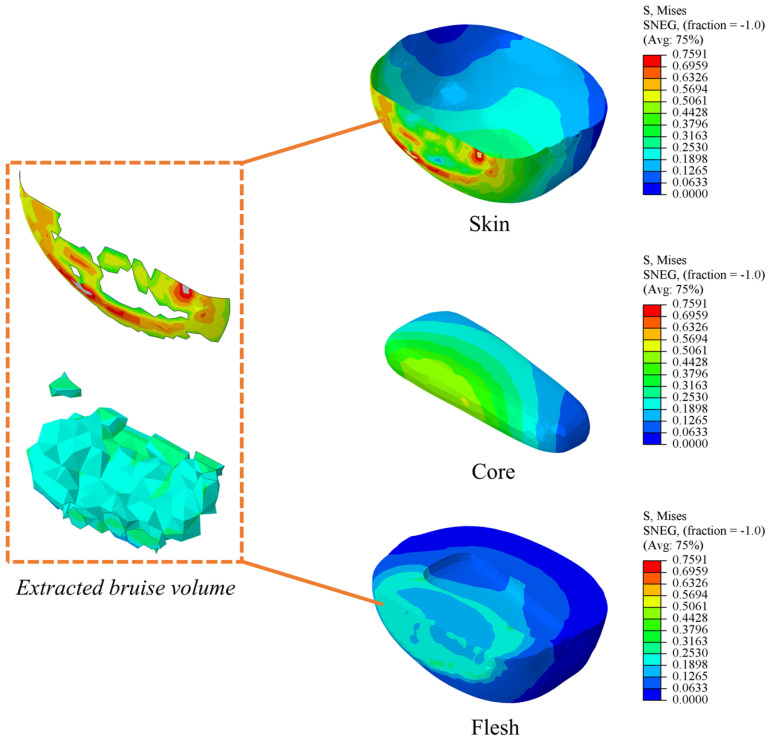
Kiwifruit bruise volume extraction.

In the process of mechanized harvesting and processing, the bruising of fruit is one of the key factors affecting its commodity value and shelf life. From the microscopic perspective, bruises are caused by the deformation and rupture of the cell wall due to excessive external pressure, which in turn leads to browning and the deterioration of the texture of pulp tissue. This damage will gradually manifest and expand during the storage and transportation of fruits. When the fruit experiences a collision, with the passage of time, the pulp tissue in the collision area will turn brown, affecting the appearance and internal quality of the fruit. In the study of the fruit bruise mechanism, mechanical parameters, such as stress [17,31], impact velocity [32], and energy absorption [33,34], were usually used by the researchers to quantify and characterize the degree of fruit bruising. However, these parameters can only indirectly reflect the damage inside the fruit but cannot directly provide the spatial distribution and volume information of the damaged area. Considering that material failure is closely related to irreversible plastic deformation, the stress distribution in the fruit during the collision process was compared with the yield stress of each tissue of kiwifruit after the fall simulation was completed. When the stress in the collision area exceeds the yield stress of the corresponding tissue, it is considered that the damage has occurred in that area. In the process of finite element simulation, the area where the stress values in skin and pulp exceed their respective yield stresses is defined as the bruised area, as shown in Figure 7. In order to intuitively analyze the damage degree of kiwifruit by falling collision, the bruise sensitivity was used as the evaluation index, which can be expressed as follows [35]:

(4) $$\mathrm{BS}=V_{b}/E_{t}$$

in the formula, *BS* is the bruising sensitivity (mm^3^ J^−1^), *V_b_* is the volume of fruit bruising, and *E_t_* is the total energy absorbed by the fruit during the collision process.

#### 2.4.6. Comparative Verification of the Finite Element Model

**Figure 8:**
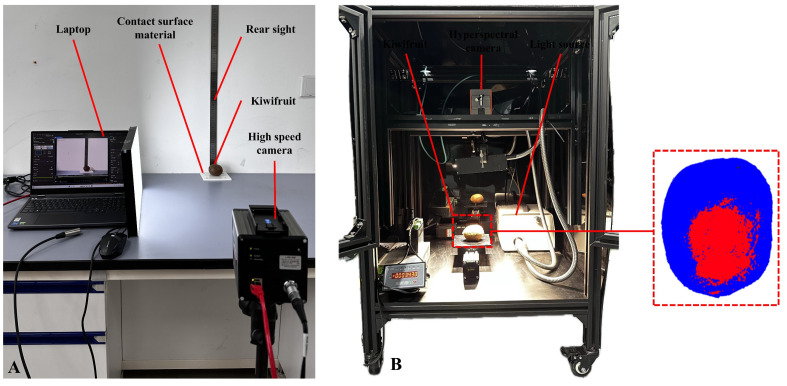
Image acquisition system. (**A**) High-speed camera system; (**B**) hyperspectral imaging system.

In order to evaluate the effectiveness of the established finite element model in the study of kiwifruit fall damage, the bruise sensitivity of kiwifruit was verified in this study through physical drop experiments, and the experimental results were compared with the simulation results. The focus of model verification is to comprehensively evaluate the dynamic behavior of kiwifruit during a falling collision and the volume and surface area of bruises generated after the collision to ensure the credibility and accuracy of the simulation results. During the experiment, kiwifruit falls freely under the action of gravity and bounces back after colliding with the predetermined contact surface material. The whole collision process can be divided into stages, such as falling, contact, separation, and rebounding to the highest point, in which the damage to the fruit mainly occurs in the contact and separation stages. Because the collision time between the kiwifruit and steel plate is extremely short, a high-speed camera system (SpeedCam Eo Sens mini-2, High-Speed Vision GmBH, Ettlingen, Germany) was used to capture the whole collision process (Figure 8A). The frame rate of the system was set to 500 frames per second, and the shooting time was 300 ms. Subsequently, image processing software was used to analyze the captured images and calculate the instantaneous speed of kiwifruit at each time point. These velocity change data were used to compare with the simulation results of the finite element model to verify the accuracy of the dynamic behavior of the model in simulating the falling process of kiwifruit. To simplify the verification process, a collision height of 0.5 m, steel plate contact material, and a collision angle of 0° were selected as the verification experimental conditions. The process of kiwifruit from contact and rebound to the highest point was captured using a high-speed camera, and its speed change was analyzed.

In the study of kiwifruit fall collision damage, accurate damage assessment presents significant challenges due to the delayed manifestation characteristics of fruit bruising. Traditional assessment methods typically involve destructive treatments, leading to irreversible damage to the specimens. Moreover, the considerable variations in bruise volume measurements across different methodologies make it challenging to accurately quantify the actual extent of fruit damage. To address these limitations and validate the accuracy of our established kiwifruit falling model, this study employed a SPECIM FX10 (Specim Spectral Imaging Ltd., Oulu, Finland) hyperspectral imaging system operating in the 400–1000 nm spectral range, with a 5.5 nm resolution. This non-destructive approach enables precise measurement of the bruised surface area of kiwifruit following impact experiments. The hyperspectral imaging system was rigorously calibrated through dark current, white reference, and spectral calibrations to ensure data reliability. Data acquisition was conducted under controlled conditions with standardized parameters (scanning speed: 30 mm/s, exposure time: 25 ms, working distance: 400 mm) in a darkroom environment to minimize external light interference. The acquired hyperspectral data underwent systematic processing using the spectral angle mapper (SAM) algorithm for bruise region classification, followed by image threshold segmentation and morphological processing to accurately delineate and quantify the damaged areas. Subsequently, the damage area measured using hyperspectral imaging was compared and analyzed with the prediction results of the finite element model to evaluate the prediction accuracy of the model. The hyperspectral imaging system is shown in Figure 8.

## 3. Results and Discussion

### 3.1. Material Properties of Kiwifruit

Through the detailed analysis and treatment of the stress–strain curves obtained from tensile and compressive tests, the key mechanical parameters of different components of kiwifruit were extracted. These parameters include Young’s modulus, the tangential modulus, biological yield stress, density, etc. Young’s modulus (Tan α) and the tangential modulus (Tan β) are the core indices reflecting the stiffness and toughness of materials when they are deformed under stress, which can reveal the response behavior of the peel, pulp, and stone under different mechanical conditions. Biological yield stress represents the maximum stress level that the fruit material can withstand when it reaches the yield point and reflects the critical conditions under which the kiwifruit begins to undergo irreversible deformation under external force. In this study, the experimentally obtained Young’s modulus and tangential modulus of kiwifruit were slightly lower than the results reported by Li et al. [36]. This discrepancy may be attributed to multiple factors, including differences in individual kiwifruit, the choice of picking time, differences in fruit maturity, and variations in testing methods. Specifically, as fruit maturity changes, the internal tissue structure of the fruit undergoes physical and chemical alterations, which in turn affect its mechanical properties. These findings provide new insights into understanding the mechanical behavior of kiwifruit and have important implications for optimizing its post-harvest handling and storage. The required material parameters in the simulation process are shown in Table 3.

### 3.2. Finite Element Simulation Results

#### 3.2.1. Grid Sensitivity Analysis

**Figure 9:**
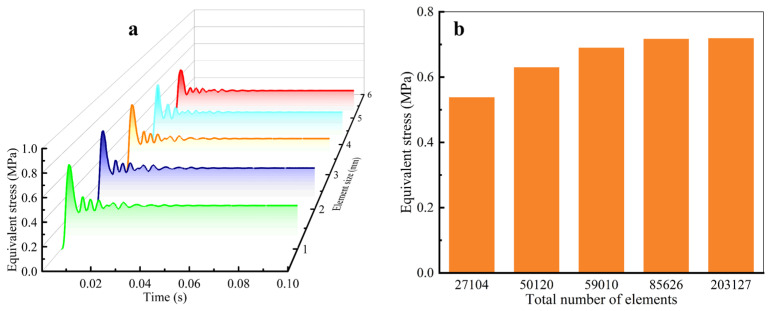
Mesh sensitivity analysis. (**a**) Effect of element size on equivalent stress; (**b**) effect of the number of elements on the equivalent stress.

In this study, a finite element numerical simulation was carried out with different grid sizes, and a grid sensitivity analysis was carried out to evaluate the influence of grid division on the simulation results, as shown in Figure 9. The analysis results showed that when the grid size was 2 mm, the maximum equivalent stress of the model changed little when the grid element size was further reduced. This indicated that when the mesh size was less than 2 mm, the mesh size had little influence on the calculation results of equivalent stress. Therefore, in this study, a grid size of 2 mm was selected as the optimal grid size in the simulation process, which can meet the requirements of calculation accuracy in terms of grid size and number. At this grid size, the finite element model consisted of 85,626 elements and 14,969 nodes.

#### 3.2.2. Model Comparison and Verification

**Figure 10:**
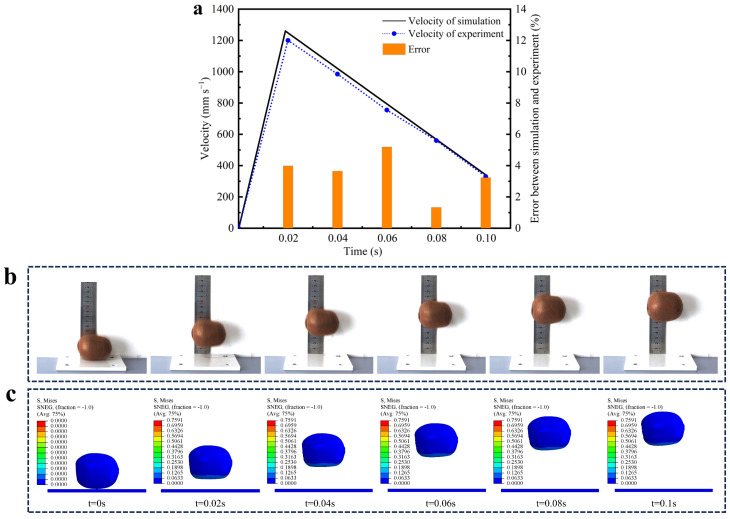
Comparison between finite element simulation and drop test. (**a**) Fruit drop velocity variation; (**b**) drop test captured by high-speed camera; (**c**) finite element simulation of the drop process.

In order to verify the accuracy of the finite element model, a comparative analysis was conducted using three aspects: the falling rebound speed, height, and bruised surface area of the fruit. This multiparameter validation method overcomes the limitations of the traditional single-method validation approach used by Hafizh et al. [37], providing a more reliable validation framework for fruit collision analysis. Firstly, the falling process of the kiwifruit was captured by a high-speed camera system, obtaining the velocity–time curve of the fruit from contact to rebound to the highest point (Figure 10a). The velocity change characteristics are similar to the results reported by Xu et al. [38] in their apple collision study but exhibit significant differences due to the unique mechanical properties of kiwifruit. The fruit velocity reaches its maximum value at the moment of contact and then gradually decreases to zero. In the separation contact stage, the velocity increases again and gradually decreases during the rebound process. Comparing the simulation results with the experimental data, it was found that the velocity change trends of the two are highly consistent, with the maximum error occurring at 0.06 s after falling and rebounding and the error value being 5.19%. This error value is within a reasonably acceptable range, indicating that the finite element model of kiwifruit drop collision and the assignment of its material parameters have high accuracy. Secondly, the rebound height of the fruit after the collision was accurately measured by a high-speed camera and compared with the rebound height predicted by finite element simulation (Figure 10b,c). The results showed that the experimental data were highly consistent with the simulation results in terms of rebound height, further verifying the effectiveness of the model. Finally, hyperspectral imaging technology was used to detect the bruised surface area of the kiwifruit after falling. The measured bruised surface area was 540.38 mm^2^, and the simulated bruised surface area was 592.46 mm^2^, with a relative error of 9.63%. The simulated value is slightly higher than the experimental value, which may be related to the weak interface bonding between the kiwifruit skin and flesh, leading to delamination and peeling during the impact process, resulting in increased energy dissipation. Despite this, the relative error between the simulation and experimental results is small, indicating that the simulation can effectively predict and evaluate the actual damage degree of the fruit.

#### 3.2.3. Finite Element Simulation Energy Analysis

**Figure 11:**
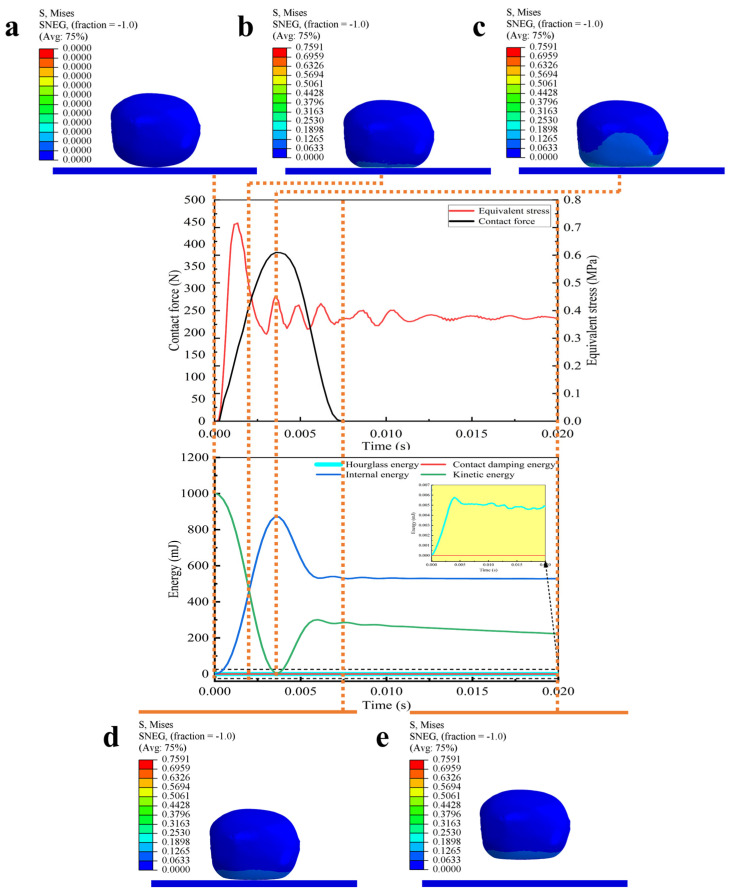
Simulation visualization output (contact surface material steel; drop height 0.5 m; collision angle 0°). (**a**) Simulation time of 0 s; (**b**) simulation time of 2.1 × 10^−^^3^ s; (**c**) simulation time of 3.7 × 10^−^^3^ s; (**d**) simulation time of 7.5 × 10^−^^3^ s; and (**e**) simulation time of 2 × 10^−^^2^ s.

To investigate the damage mechanism of kiwifruit during the falling collision, the finite element simulation method was used to systematically analyze the effects of different falling heights, contact materials, and collision angles on kiwifruit damage. By processing the simulation results, the changes in the contact force and equivalent stress of kiwifruit during falling were obtained. In order to extract the damaged area of fruit, the Von Mises failure criterion was used to analyze the equivalent stress. The area where the stress exceeded the biological yield stress threshold of pulp by 0.26 MPa was defined as the pulp damage area, and the area where the stress exceeded the peel yield stress threshold of 0.52 MPa was defined as the peel damage area. Figure 11 shows the changes in contact force, equivalent stress, and energy of kiwifruit peel under the working conditions of a falling height of 0.5 m, a steel plate contact surface material, and a collision angle of 0°.

The results showed that the collision occurred at 0 s of the starting moment of the simulation. Subsequently, the kinetic energy of kiwifruit was quickly converted into internal energy and contact energy. When the simulation time reached 3.7 × 10^−3^ s, the contact force between the kiwifruit and steel plate reached the peak, and the equivalent stress on the fruit also reached the maximum, indicating that the kiwifruit absorbed the largest amount of collision energy. At 7.5 × 10^−3^ s, the kiwifruit began to separate from the contact surface. However, even after the collision, the equivalent stress of the kiwifruit had not completely recovered to its initial state, indicating that residual stress had been generated in the kiwifruit due to the plastic deformation caused by the collision. This residual stress may lead to the deterioration of fruit quality during subsequent storage and transportation and become one of the potential factors affecting fruit quality. This finding is similar to the conclusion drawn by Du et al. [39] in their study on bruise detection in fruits and vegetables, in which they discovered that the plastic deformation and residual stress caused by the impact are significant factors leading to the deterioration of fruit quality. In the study of falling, hourglass energy is a kind of numerical dissipation energy in the finite element model, mainly appearing in the non-physical deformation process of the elements, that is used to reflect the stability of the model and the accuracy of calculation [40]. Research showed that in order to ensure the reliability of simulation results, the hourglass energy should not exceed 5% to 10% of the internal energy [41]. Based on this evaluation criterion, the energy distribution of all simulated scenes was analyzed in detail. The results showed that the hourglass energy did not exceed the recommended range, and the highest value did not exceed 0.08% of the internal energy. Therefore, the established finite element model had high rationality and accuracy in energy distribution.

#### 3.2.4. Equivalent Stress and Contact Force of Fruit Falling

**Figure 12:**
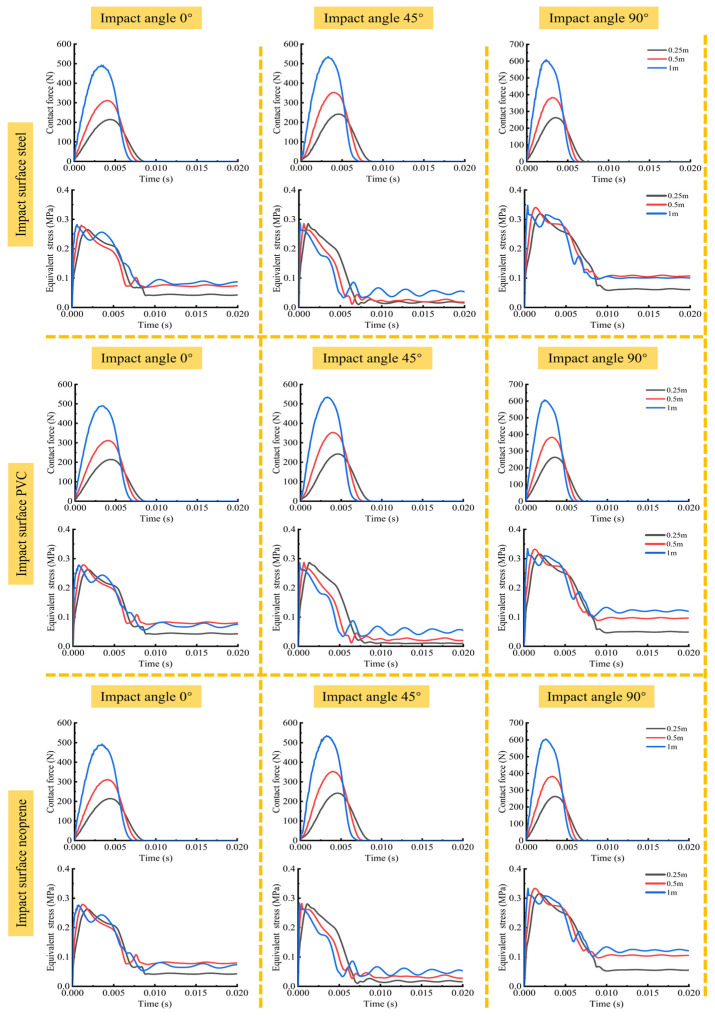
Simulated contact force and equivalent stress under different drop conditions.

In the study of the drop collision mechanical behavior of kiwifruit, the contact surface material, collision angle, and falling height are important factors affecting the collision response. Through the post-processing of the simulation results, the variation curves of contact force and equivalent stress of kiwifruit pulp with time in different falling scenarios were obtained (Figure 12). The figure shows the changes in contact force and equivalent stress of kiwifruit colliding with different contact materials (steel, PVC, and neoprene), at different collision angles (0°, 45°, 90°), and from different falling heights (0.25 m, 0.5 m, and 1 m). For the same contact surface material and falling height, the contact force and equivalent stress of fruits at different collision angles showed significant differences. Specifically, as the falling angle increased from 0° to 90°, the contact force and the peak equivalent stress of the fruit increased. When the contact surface material was steel, the contact force reached a peak value of 492.99 N at a collision angle of 0° and a drop height of 1 m and increased to 607.82 N at a collision angle of 90°. This phenomenon can be explained as follows: at a small collision angle, the contact area between the fruit and the contact material was maximized, the impact force was distributed in a large range, the energy was effectively dispersed, the concentration effect of instantaneous impact force was reduced, and the local stress concentration was reduced. The change trend of equivalent stress was consistent with the contact force. When the collision angle was 0°, the peak value of equivalent stress was the smallest, and it gradually increased with the increase in angle. On a steel contact surface, the peak value of equivalent stress was 0.28 MPa at a 0° collision angle, and it increased to 0.34 MPa at a 90° collision angle. When the fruit collided with the contact material at an angle of 45°, the contact area between the fruit and the contact material increased, and the impact force was dispersed compared with the state of falling at an angle of 90°. The impact force can be divided into two directions: along the fruit axis and perpendicular to the fruit axis. Part of the kinetic energy will be converted into vertical rotation and horizontal sliding. Therefore, the vertical contact force was reduced compared with the 90° collision, the equivalent stress of fruit tissue was reduced, and the damage after the collision was relatively reduced.

This indicated that there was a relatively large contact area between the fruit and the contact surface material at a small collision angle, which can effectively disperse the stress and reduce the mechanical damage to the fruit. At the same collision angle and falling height, different contact surface materials have relatively little influence on the contact force and equivalent stress of fruits. This is mainly because the elastic modulus of the contact surface material was much greater than that of the kiwifruit, resulting in greater deformation of the fruit in the collision process than that of the contact material. In the process of short-term collision, the bio-mechanical properties of kiwifruit materials played a decisive role. The deformation and energy of the fruit were mainly transmitted through the internal tissue, which can absorb and buffer most of the impact in a short time. The stress was mainly concentrated at the moment of contact.

Therefore, in order to reduce the collision damage of fruits during harvesting and post-processing, factors such as the maturity, hardness, and size of fruits should be comprehensively considered, and cushioning materials matching the mechanical properties of fruits should be selected. By reducing the elastic modulus of the cushioning material, it can absorb and disperse more impact energy under stress, reduce the deformation and stress concentration of the fruit, and thus effectively reduce the risk of damage to the internal tissue of the fruit. This is consistent with the studies of Lin et al. [42] and Cevher et al. [43], who emphasized the importance of selecting appropriate cushioning materials during post-harvest handling to reduce impact damage to fruits.

In addition, the drop height had a significant influence on the contact force and equivalent stress. With the drop height increasing from 0.25 m to 1 m, the peak contact force and equivalent stress increased significantly, which was in line with expectations. The reason was that the higher falling height made the fruit have more kinetic energy, which was quickly transformed into stress and deformation energy at the moment of collision. The increase in falling height also shortened the time for the contact stress to reach the maximum value, indicating that the collision at a higher falling height was more intense, the energy was released faster, and the fruit withstood a greater load in a short time. This finding is consistent with the results of Pathare et al. [44], who studied the effect of drop height on the impact damage of pear fruits and found that the impact force and degree of damage increased significantly with increasing drop height.

#### 3.2.5. Prediction of the Sensitivity of Bruises Using the Multiple Regression Method

In this study, the finite element numerical simulation method was used to simulate the falling damage of kiwifruit in order to explore the effects of different falling heights, collision angles, and contact surface materials on the fruit damage. Through the post-processing of the simulation results, the damage volume data of the fruit were extracted, and the bruise sensitivity value of the fruit was calculated. On this basis, referring to the research methods of Xu et al. [17] and Guan et al. [31], the response surface model of damage sensitivity was constructed using multiple regression analysis. In order to predict the bruise sensitivity of kiwifruit when it collided with different contact materials, based on the response surface method (RSM), the prediction model of bruise sensitivity using different contact materials was as follows:

$$\left\{ \begin{array}{l} Z_{1}=1419.5306+4039.4034X+0.2218Y-4.4657XY+956.2515X^{2}+0.0843Y^{2}\\ Z_{2}=1021.1223+4839.5683X+2.5997Y-2.1677XY+42.4485X^{2}+0.0132Y^{2}\\ Z_{3}=-404.2321+8672.6431X+0.3499Y-0.5917XY-3093.3563X^{2}+0.0076Y^{2} \end{array}\right.$$

**Figure 13:**
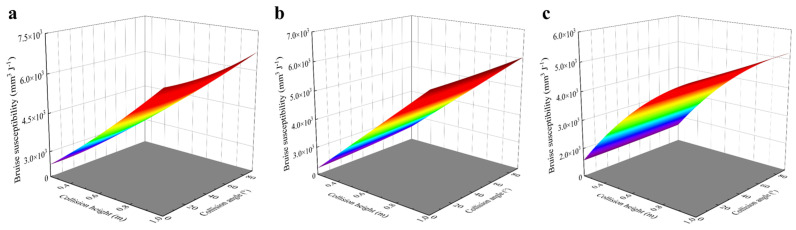
Response surface of fruit bruise susceptibility. (**a**) Steel impact material; (**b**) PVC impact material; and (**c**) neoprene rubber impact material.

Among these, *Z*_1_, *Z*_2_, and *Z*_3_ respectively represented the bruising sensitivity (mm^3^ J^−^^1^) of kiwifruit when it collided with steel plates, PVC, and chloroprene rubber surfaces, X was the falling height (m), and Y was the collision angle (°). Through statistical analysis, it was found that when the collision materials were steel plate and PVC, the falling height had a very significant effect on the bruise sensitivity of kiwifruit (*p* < 0.01), the collision angle reached a significant level (*p* < 0.05), and the collision degree was in the order of falling height > impact angle. When the impact material was neoprene, the impact height was very significant (*p* < 0.01), but the collision angle was not significant (*p* > 0.05). In order to visually show the influence of experimental factors and their interaction on the bruise sensitivity of kiwifruit, the response surface diagram of kiwifruit when it collided with the surfaces of steel plate, neoprene, and PVC was drawn (Figure 13). The results showed that the bruise sensitivity of kiwifruit increased significantly with the increase in falling height, especially on the steel plate and PVC surfaces. When the collision surface material was a steel plate, the falling height was 1 m, and the collision angle was 90°, the damage sensitivity of kiwifruit was 6716.07 mm^3^ J^−^^1^ at the maximum. When the impact surface material was neoprene, the falling height was 0.25 m, and the impact angle was 0°, the damage sensitivity of kiwifruit was 1570.59 mm^3^ J^−^^1^ at the minimum. In addition, the change in collision angle will also affect the damage degree of kiwifruit, but its influence degree is lower than the change in falling height. Through response surface analysis, the judging coefficients (R^2^) of the bruise sensitivity of kiwifruit when it collided with steel plate, chloroprene rubber, and PVC surfaces were 0.993, 0.995, and 0.980, respectively, which showed that the constructed response surface model had high fitting accuracy.

### 3.3. Limitations and Future Application Research

Although the prediction model of kiwifruit bruise sensitivity was established in this study using a finite element simulation and response surface method, and the applicability and reliability of the model were verified, there are still some limitations that need further discussion. First of all, the material properties of the kiwifruit components were obtained through compression and tensile tests, and there may be some experimental errors in the determination of material properties for different varieties and maturity levels of kiwifruit. These errors may affect the accuracy of the finite element model, thus affecting the accuracy of damage prediction. Therefore, the effect of different maturity fruits on fall bruising needs to be investigated further in future studies. Meanwhile, more precise and scientific methods, such as nano-indentation technology and dynamic mechanical analysis, can be used to determine the material properties of each component of fruit to improve the accuracy of the model. Secondly, in this study, only the effects of three falling heights, three collision angles, and three contact materials on fruit falling damage were considered. However, in the actual picking and processing process, the bruising of kiwifruit may also be affected by factors such as temperature, humidity, and fruit hardness. Therefore, it is necessary to further improve the prediction model of bruise sensitivity in future research, consider more factors that affect fruit damage, such as different varieties, maturity, and temperature conditions, and use multivariate statistical methods, such as principal component analysis and cluster analysis, to reveal the mechanism of kiwifruit fall damage under the comprehensive action of multiple factors. Finally, the established prediction model was based on samples of specific varieties and maturity, and its application scope may be limited. There are differences in physical and mechanical properties and tissue structure of different varieties of kiwifruit, which may lead to deviation in the prediction results of bruise sensitivity. Therefore, it is necessary to carry out more extensive research on the applicability of varieties to expand the application scope of the model.

## 4. Conclusions

In this study, aiming to address the issue of kiwifruit being vulnerable to mechanical damage in the process of mechanized picking and processing, which affects its commodity value and shelf life, the objective was to improve the harvest quality of fruit by accurately predicting the bruising. The mechanical properties of materials of various components of kiwifruit were measured through compression and tensile tests, and a multiscale finite element model based on the macro-structure of kiwifruit was established to simulate the bruise sensitivity of the fruit in the process of falling collision, which effectively solved the problem that it was difficult to visualize the impact damage of the fruit in traditional physical experiments. In this study, the effects of falling height, collision angle, and contact surface materials on kiwifruit bruises were also deeply discussed, and an empirical model for predicting bruise sensitivity was constructed for different contact surface materials. The main conclusions were as follows:

(1) The simulation results were in good agreement with the experimental data, with a maximum error value of 5.19% in speed variation, indicating that the finite element model of kiwifruit drop collision and its material parameter assignment had high accuracy and credibility.
(2) In all simulation scenarios, the hourglass energy did not exceed the recommended range, with the maximum value of hourglass energy not exceeding 0.08% of the internal energy, which showed that the established multiscale finite element model of kiwifruit had good rationality and accuracy in energy distribution and ensured the reliability of the simulation results.
(3) The contact force and equivalent stress of kiwifruit were influenced by the contact surface material, collision angle, and falling height. On the contact surface material with high rigidity, a higher falling height and larger collision angle will increase the risk of kiwifruit bruises. The sensitivity of fruit bruises increased significantly with the increase in falling height and was more pronounced on steel plates and PVC surfaces. When the contact surface material was a steel plate, the falling height was 1 m, and the collision angle was 90°, the maximum bruise sensitivity of kiwifruit reached 6716.07 mm^3^ J^−1^. However, when the contact surface material was neoprene, the falling height was 0.25 m, and the collision angle was 0°, the damage sensitivity was the lowest, at 1570.59 mm^3^ J^−^^1^.
(4) In the process of fruit picking and post-processing, it is recommended to use materials with a low elastic modulus in contact with the fruits while minimizing the height difference between the fruit and the collecting device and avoiding collisions between the fruits and rigid materials at a collision angle of 90° to reduce the occurrence of mechanical damage.

In this study, an important theoretical basis and technical support for kiwifruit in the process of picking and post-processing were provided, which were helpful in reducing fruit damage and prolonging the shelf life of fruit. At the same time, the established multiscale finite element model and research method can also provide useful references for the damage prediction and protection research of other similar fruits.

## Figures and Tables

**Table 1:** Geometry of kiwifruit reverse modeling sample.

Dimensions	Unit	Model Measurement
Fruit Length (*L*)	mm	64.18
Maximum diameter of the equatorial section (*D_max_*)	mm	53.46
Minimum diameter of the equatorial section (*D_min_*)	mm	50.72
Surface area	mm^2^	10,182.14
Volume	mm^3^	195,931.16

**Table 2:** Mesh size and element count for the kiwifruit model.

No.	I	II	III	IV	V
Element size (mm)	1	2	3	4	5
Elements	203,127	85,626	59,010	50,120	27,104
Nodes	35,843	14,969	10,245	8266	4704

**Table 3:** Mechanical property parameters of kiwifruit and collision surface materials.

Materials	Young’s Modulus (MPa)	Tangent Modulus (MPa)	Bio-Yield Stress (MPa)	Density (kg m^−3^)	Poisson’s Ratio
Skin	10.69 ± 0.46	—	0.53 ± 0.12	960 ± 30	0.30
Flesh	1.57 ± 0.12	0.92 ± 0.06	0.26 ± 0.07	1030 ± 45	0.40
Core	5.11 ± 0.28	0.83 ± 0.04	1.12 ± 0.23	1120 ± 70	0.40
Steel	2.1 × 10^5^	—	235	7850	0.33
PVC	70	—	—	60	0.30
Neoprene	7.8	—	—	930	0.47

## Data Availability

The original contributions presented in this study are included in the article, and further inquiries can be directed to the corresponding author.
